# Undergraduate student attitudes towards animal welfare science: An investigation to inform teaching approaches

**DOI:** 10.1017/awf.2025.10032

**Published:** 2025-08-19

**Authors:** Annabelle Beaver, Beth Ann Ventura

**Affiliations:** 1 Animal Behaviour and Welfare Research Group, Animal Science Research Centre, Harper Adams University, Newport, Shropshire TF10 8NB, UK; 2 Large Animal Clinical Sciences, College of Veterinary Medicine, Michigan State University, East Lansing, MI, USA; 3 Department of Life Sciences, University of Lincoln, Brayford Pool, Lincoln LN6 7TS, UK

**Keywords:** Action research, animal ethics, animal welfare, pedagogy of animal welfare, science, student beliefs, student viewpoints, undergraduate attitudes

## Abstract

The study of animal welfare is essential for undergraduates seeking to pursue careers with animals, yet pedagogical research on this topic is limited. While animal welfare is an accepted (albeit relatively new) scientific discipline, student views on animal welfare as a science require further exploration. This article reports the findings from a mixed-methods action research project undertaken at Harper Adams University (HAU) in the UK. Undergraduate student questionnaire responses (n = 123) revealed key attitudinal constructs related to animal welfare, and relationships to demographic factors. Students overwhelmingly defined animal welfare in terms of health; however, rural (compared to urban) students more often perceived ‘naturalness’ as important in the maintenance of good welfare. Notions of what constitutes good animal welfare appeared to be mediated by prospective career paths. For instance, veterinary nursing students were more likely to define animal welfare based upon resource-based measures and appropriate treatment of animals, which may link to their future role in educating clients on these topics. Finally, student attitudes toward animal welfare science revealed deeper epistemological views on the meaning of ‘science’. That is, natural sciences were seen as trustworthy; students invoked the Scientific Method and disciplines such as neurobiology to bring credence to animal welfare science. Conversely, aspects of animal welfare addressed by the social sciences were dismissed as unscientific. Based on these results, recommendations for action are proposed, which include further research into the attitudes of educators, strategies for engaging with dissatisfied student groups, and elevating the social sciences within animal welfare curricula.

## Introduction

A strong foundation in animal welfare science is essential for undergraduates pursuing diverse career paths with animals. Students in animal-focused courses may have significant future influence on animal welfare decisions across a broad range of sectors. Given the growing number of animal-focused courses in the UK (see MacKay *et al.* 2020), coupled with a rising global interest in animal welfare, further research around the delivery and teaching of the subject within the UK higher education context is needed. This research was conducted at a university in the UK, and the terms used throughout the article are standard in British higher education. A list of these included terms and definitions are shown in Table 1.

**Table 1.** Glossary of terms and definitions as they are used in this Welfare Science Exploratory Factor Analysis

Term	Definition
Course	A ‘course’ in British universities is equivalent to a full degree programme, such as a Bachelor’s degree. Students typically enter a named course in their first year of study (e.g. Animal Health and Welfare), which has a pre-defined focus
Module	A ‘module’ in the context of British higher education refers to a self-contained unit of study within a larger academic programme or ‘course’ (see above). A module typically focuses on a specific topic or subject area within the student’s broader field of study. Modules usually have a set number of credit points associated with them, which contribute towards the overall degree
Lecturer	In the UK, a ‘lecturer’ is a professional (i.e. member of the academic faculty) involved in teaching and/or research at a higher education institution. A UK lecturer differs from a lecturer in North American contexts in that the latter is not typically appointed to conduct research. Although a UK lecturer is roughly equivalent to an assistant professor in North America, the collective term ‘lecturers’ will be used throughout to refer to UK higher education professionals at all ranks

A substantial body of research has examined attitudes towards animals and animal welfare among students in a variety of degree programmes, including animal science undergraduates, veterinary students, and those in pre-university programmes (e.g. Paul & Podberscek 2000; Furnham *et al.* 2003; Heleski & Zanella 2006; Lord *et al.* 2010; Hazel *et al.* 2011; Mazas *et al.* 2013; Colombo *et al.* 2016; Ostović *et al.* 2016; Çavuşoglu & Uzabaci 2021; Platto *et al.* 2022; Sullivan *et al.* 2022; Robbins *et al.* 2021 Brunt *et al.* 2024). Broadly, this literature has generated understanding of student attitudes, empathy, and perceptions surrounding ethical behaviour towards different animal species. However, what is less understood is how students perceive the area of animal welfare *as a topic of study* (with some exceptions, e.g. see Johnson *et al.* 2020).

While there is broad acknowledgement that animal welfare education is valuable, primary empirical research on instructional design or pedagogic approaches to the topic have been less well developed (MacKay 2020). Much of the focus has been directed to training in the veterinary context (e.g. Broom 2005; Hewson *et al.* 2005; Molento & Calderon 2009), culminating in the presentation of a model veterinary curriculum in animal welfare (Lord *et al.* 2017). In response to this, the last decade has seen a proliferation of studies addressing the training of day-one competencies related to animal welfare (e.g. De Briyne *et al.* 2020; Johnson *et al.* 2020; McGreevy *et al.* 2020; Ventura *et al.* 2021; Proudfoot & Ventura 2021; Jones *et al.* 2024) so as to better prepare veterinarians for real-world welfare challenges (De Paula Vieira & Anthony 2020).

Relatively fewer studies have addressed undergraduate attitudes towards the teaching of animal welfare, with existing research often focusing upon whether students think animal welfare merits inclusion in the curriculum. For example, Pejman *et al.* (2023) examined the extent to which secondary and university students in eight EU countries were receptive to the topic of animal welfare. The authors determined that acceptance depended on several factors, including gender, ethical beliefs, country of origin, and level of study. Mijares *et al.* (2021) identified specific welfare-related subtopics of interest to undergraduate and graduate students in animal science programmes in the United States, concluding that the majority supported the inclusion of animal welfare within the curriculum.

To the authors’ knowledge, few studies have considered student attitudes toward animal welfare *as a scientific discipline*, or how student backgrounds and broader epistemological frameworks may influence how they define and understand animal welfare as a science. This may be because animal welfare science is a comparatively new and rapidly developing field (Broom 2005; Mellor *et al.* 2009). Marchant-Forde (2015; p 16) chronicles the history of the discipline, describing how the science has arisen from the cross-fertilisation between disparate subjects: [Animal welfare science] *has emerged into a truly multi- and inter-disciplinary science, encompassing… behavior, physiology, pathology, health, immunology, endocrinology, and neuroscience, and influenced by personal and societal ethics.*

It has also been argued that education research should be more purposefully included within this multidisciplinary space (MacKay *et al.* 2020). Thus, we sought to explore the ways in which undergraduate students in animal-focused courses at a UK public university conceptualised animal welfare science. We also explored how student backgrounds, demographics, and prospective career paths may influence perceptions of their animal welfare modules. These investigations were carried out using the framework of action research; in higher education, action research aims to improve scholarship in teaching and learning and contribute to educational theory (Arnold & Norton 2018). It is hoped that these data will enable consideration of how lecturers and students may work together to modify or enhance teaching approaches to increase interest and perceived importance of animal welfare content.

## Materials and methods

### Conceptual framework

**Figure 1.**
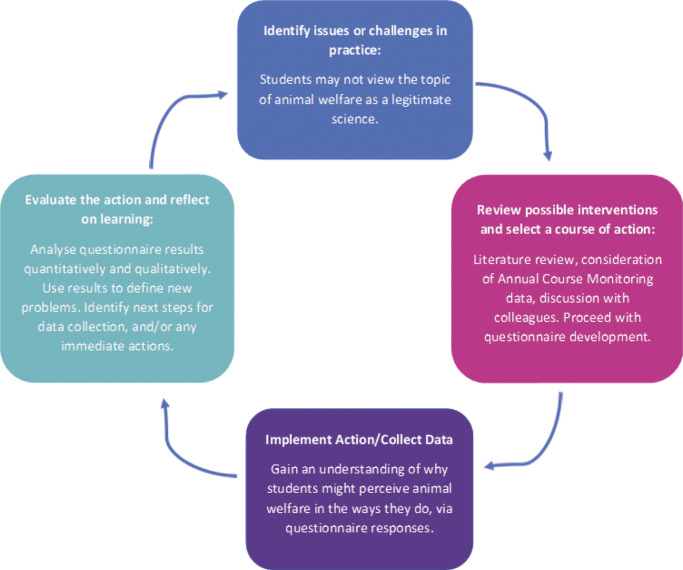
The stages of the Action Research process as undertaken within the current study to evaluate UK undergraduate students’ conceptions of the scientific discipline of animal welfare. Adapted from Arnold and Norton (2018).

We have drawn upon several tenets of Participatory Action Research (PAR) to guide the present study. PAR is an inclusive research process that emphasises the lived experiences of participants, fosters critical reflection, and elevates collaboration between participants and researchers to effect change (Lloyd-Evans *et al.* 2023). Using Arnold and Norton’s (2018) Action Research Cycle framework, we delineate the core research stages (Figure 1). These stages were developed by AB at study outset and have been iteratively modified to reflect new directions arising from the action-reflection process (McNiff 2017).

### Study aims and objectives

The overarching objective of this research was to explore the ways in which undergraduate students in animal-focused courses conceptualise animal welfare as science and as a topic of study. The questionnaire items were designed to enhance this understanding as much as possible within this local context. Thus, to understand whether students considered animal welfare to be a science, we thought it critical to understand their perspectives on the meaning of ‘science’ and their relationship to science. We were also interested in learning more about how students defined animal welfare, and their knowledge and perceived knowledge of the topic.

Our *a priori* research objectives for the quantitative analysis focused more specifically on the relationships between student attitudes and demographic factors. In particular, we aimed to understand whether students in different course groups differed in their knowledge and attitudes toward animal welfare and the legitimacy of animal welfare science. We also sought to uncover latent factors that may explain these student viewpoints and assess their relationship with student background (i.e. rural farm, rural non-farm, or town/city), sphere of welfare prioritised, gender, and course group. See Table S1 (Supplementary material_1) for a numbered list of *a priori* research questions.

### Researcher positionality

This article seeks to explore student attitudes to animal welfare as a scientific discipline and as a field of study. As authors, we therefore acknowledge our own views of this field to support transparency and trustworthiness in the research process (Holmes 2020). As articulated by Marchant-Forde (2015), and as animal welfare scientists ourselves, we consider animal welfare to be a legitimate scientific field that is both multi- and inter-disciplinary, drawing from both within and outside the natural sciences. We conceive that animal welfare itself relates to the internal state of an animal, encompassing both physical and mental well-being, and concerning “*its attempts to cope with its environment*” (Broom 1986; p 524). Thus, the welfare of an animal is an objective reality for that animal; as scientists, we can attempt to gain insights into the animal’s welfare state by using validated proxy measures that are components of, or at least co-vary with, welfare. However, as with any science, the topics we consider worthy of research are societally dictated and culturally determined (Fraser *et al.* 1997). Moreover, biological scientific findings alone are often insufficient for widescale animal welfare improvements, due to complex influences of social norms and values. From this perspective, we view social science research as a critical component of animal welfare science, facilitating the translation of natural research findings into practice. We approach our work acknowledging that scientific questions about animal welfare can often be best addressed by integration of both natural- and social-science methods, as an understanding of societal paradigms (by means of social science investigation) plays a fundamental role in our ability to meaningfully improve animals’ welfare states.

### Questionnaire development

Development of the questionnaire was guided by previously validated scales assessing student attitudes towards science (Kind *et al.* 2007) and animal welfare (Mijares *et al.* 2021). An informal review of 2020–2022 Course Monitoring Data from animal welfare modules was also conducted to inform questionnaire content. At the end of each academic year, Harper Adams University (HAU) gives students the opportunity to complete a module-specific online questionnaire regarding their experience. Students can provide quantitative feedback on the module (e.g. by responding to Likert-type questions) in addition to free-text responses. Upon request, the feedback for a specific module is sent to those who contribute to the teaching of this module. Items were developed using seven-point Likert items ranging from ‘strongly disagree’ to ‘strongly agree’. Conceptual frameworks including the Five Freedoms (Farm Animal Welfare Council 1979) and three spheres (‘health’, ‘affective state,’ and ‘natural living’; Fraser *et al.* 1997) were used to inform the development of open-ended and ranking questions.

The questionnaire was created using Jisc Online Surveys and face validity of items (Gaber 2010) was assessed by six university educators in animal welfare. Based upon this expert assessment, three statements were reworded, four were dropped, and six were added. The questionnaire was pilot-tested (n = 10) among students prior to launch, which led to minor amendments to the phrasing of two Likert statements. The final questionnaire consisted of 20 Likert-item questions, seven demographic questions, and seven open-ended and ranking questions (see Supplementary material_2). The questionnaire was broadly divisible into four main sections. Within each section, we have referenced the specific objective or objectives that the questions were designed to address:

*Section 1*

Evaluated whether students considered animal welfare to be a legitimate scientific discipline (Qs 1, 2, and 3 [items 6–8]) and their views on, and relationship to, science in general (Q3 [items 1–5]).

*Section 2*

Addressed students’ views on the value of their animal welfare curriculum and perceived knowledge of the topic (Q4 [items 1–11]).

*Section 3*

Examined student definitions of animal welfare (Q5 and 6) and their actual knowledge of animal welfare (Q7). Three additional questions (8–10) were included to gain more qualitative information regarding the teaching of animal welfare at HAU; these responses often addressed specific modules and lecturers and were intended for internal use only as part of wider action research efforts.

*Section 4*

The final section of the questionnaire collected demographic information, such as course, year, gender, age, and background (urban, rural non-farm, or rural farming; Q11–17).

### Ethical approval

Ethical approval was obtained prior to study initiation via delegates from the HAU Ethics Committee (approval granted for named project) and no personal information was collected from participants. Students were made aware of the questionnaire during their animal welfare modules but were advised that participation was optional (and untraceable). No module credit or marks were associated with completion of the questionnaire.

### Research context, participants, and recruitment

Data collection occurred between November 2022 and February 2023, and the target population consisted of undergraduates at all year levels (year 1–4) who had taken, or were currently taking, at least one animal welfare-focused module at HAU. HAU is a public university providing specialised higher education across rural and agricultural sectors. The university considers the inclusion of animal welfare science into the undergraduate curriculum to be essential for students whose future work will involve animals. The students taking part in the questionnaire were enrolled in the following courses: Animal Behaviour and Welfare (ABW), Animal Health and Welfare (AHW), Animal Production Science, Bioveterinary Science, Veterinary Physiotherapy (Vet Physio), Veterinary Nursing (Vet Nursing), Wildlife Conservation, and Zoology. HAU module leaders were contacted via email to share the questionnaire with their cohorts. Modules included: Introduction to Animal Welfare, Behaviour & Ethics (first year); Principles of Animal Behaviour & Welfare (second year); Applied Companion Animal Health, Welfare & Behaviour (fourth year). Students in their third year at HAU typically complete a placement year, spending a year working or volunteering with a specific company or organisation (see Placement). Relevant HAU placement managers were also contacted to share the questionnaire link with students out on placement. Students were provided 15 min of class time (either at the beginning of class or during a mid-class break) to complete the questionnaire, a copy of which was also posted on module homepages.

### Analysis

#### Statistical analysis

Descriptive statistics pertaining to student demographics (e.g. course, year of course, age, and gender) and attitudes (e.g. which welfare sphere was prioritised) were conducted in SAS (v 9.4) using PROC UNIVARIATE and PROC FREQ for continuous and categorical variables, respectively. Relevant figures have been produced using Canva (v 1.38.0).

Inferential statistics were also conducted in SAS, with significant relationships defined at *P* < 0.05. Due to ordinal outcome variables, Kruskal Wallis tests (PROC NPAR1WAY) were used to examine whether there was an overall difference between course groups in the extent to which students: (1) considered animal welfare to be a science; and (2) believed they already knew everything about animal welfare. A significant omnibus test was followed up with Bonferroni-corrected Mann-Whitney *U* tests to compare each course group to the ABW cohort. Due to the small sample size of AHW and Animal Production Science students, these students were combined with Bioveterinary Science to form a group referred to as ‘Other Animal Science courses’. We also aimed to assess whether different courses and different student backgrounds (i.e. rural farm, rural non-farm, or town/city) were associated with the welfare sphere prioritised by students (health, affective state, or natural living). Due to several expected cell counts below 5, Fisher’s Exact Tests (PROC FREQ) were conducted to evaluate this question.

Students were asked to name as many of the Five Freedoms as they could off the top of their heads. Following tests for normality on the dependent variable (PROC UNIVARIATE), an ANOVA (PROC ANOVA) was conducted to evaluate whether the number of correctly identified freedoms varied by course group. A significant result was followed up by Tukey corrected *post hoc* tests of mean differences.

Exploratory factor analysis was performed using SPSS (v 27). Factor analysis condenses a collection of variables into a smaller number of factors that summarise the key relationships. ‘Exploratory’ factor analysis (EFA) is employed to discover summary constructs when the underlying theories surrounding the structure of the variables is unknown (Goldberg & Velicer 2006). Two separate EFAs were conducted to evaluate attitudes toward teaching of animal welfare at HAU, and attitudes toward animal welfare and science in general. These EFAs are hereafter referred to as ‘the Teaching EFA’ and ‘The Welfare Science EFA’. Although some have argued that seven-point ordinal variables can effectively be treated as continuous, interval data remain a key assumption of factor analysis. We therefore elected to monotonically transform all variables using the optimal scaling approach (CATPCA; Meulman *et al.* 2004; Linting & van der Kooij 2012) prior to conducting the EFAs. The spline ordinal transformation was applied (degree = 2; interior knots = 2). The EFAs were then conducted using the Maximum Likelihood approach for factor extraction, and factor weightings were orthogonally rotated using the Varimax rotation method. Any attitudes that had a rotated loading of < |.4| on all factors were removed from the analysis, and the EFAs repeated. Factors with eigenvalues of ≥ 1 were retained as final factors. Relevant figures have been produced using SPSS.

Each resulting factor was then used as the outcome variable in multivariable linear regression models (SAS; PROC GLM), with relevant demographic variables and factors from the other EFA included as predictors. Any highly correlated variables (r ≥ 0.7) were not included together in any models but were first assessed in univariable analyses and for contextual relevance. Final models were generated using a backwards stepwise elimination procedure, and all variables retained in the final models were significant at the *P* < 0.05 threshold. Relevant *post hoc* comparisons were undertaken using the Tukey adjustment method.

Following the thematic analysis (see below) of student definitions of animal welfare and views of animal welfare as a science, select themes were assessed according to course group using Chi-squared tests (PROC FREQ).

#### Thematic analysis

A constructionist thematic analysis (Braun & Clarke 2006; Kiger & Varpio 2020) was carried out to describe both semantic (i.e. explicit) and latent themes within the data. We used the iterative six-phase analytical process outlined by Braun and Clarke (2012) as a guide, beginning with a phase of data familiarisation. In this first phase, qualitative responses to questionnaire items were read and re-read, and notes were taken regarding potential overt themes and salient quotations. Statements were also grouped using Excel® into these potential themes, with the goal of gaining a degree of insight into the broader data landscape.

In phase two, a more systematic approach was taken in the development of initial codes (both semantic and latent). A data-driven, inductive process was first used to open-code the data and understand meaning communicated by the students. All responses from students were assigned to at least one code, and NVivo software (QSR International, Cheshire, UK) was used to organise the codes. Following this first round of coding, deductive approaches were employed, which centred around theoretical frameworks for conceptualising animal welfare. These included the Three Spheres of welfare (Fraser *et al.* 1997), the Five Domains model integrating human-animal interactions (Mellor *et al.* 2020), and DAWCon (Arndt *et al.* 2022) which considers animal-based measures, internal states, external resources, events, and animals’ ability to adapt.

Phase three involved generation of candidate themes, in which codes were retained, deleted, combined, or collapsed according to shared meaning. For instance, when asked to provide a definition for animal welfare, one student responded “*a really interesting topic*”; the code containing this quotation was deleted because it did not fit a broader theme as defined by the researchers. Similarly, codes relating to subjectivity, absence of objectivity, opinions, and emotionality were condensed into a single theme, as were the codes relating to data, facts, the scientific method, and peer-reviewed research.

In phase four, the candidate themes were examined for internal consistency and refined or pared down to support a coherent data narrative.

Phase five was conducted iteratively throughout the process: some themes were reclassed as subthemes, relevant quotations were selected, cropped, and reordered to support each theme or subtheme. The write-up of the results and discussion (phase six) also occurred iteratively. Excerpts from student responses to free-text questions are included in the reporting of results to enhance research credibility and ensure student voices remain central to the process (Creswell & Poth 2018).

## Results

### Demographics

**Figure 2.**
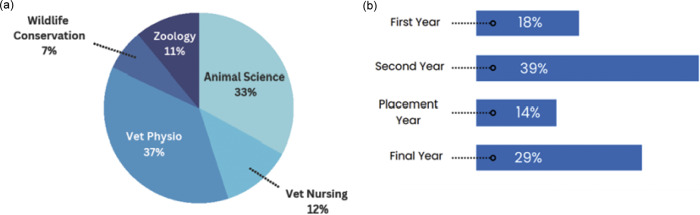
The distribution of (a) participating courses and (b) participating course years among UK undergraduate students surveyed to evaluate conceptions of animal welfare science.

One hundred and twenty-three undergraduate students were included in the final analysis, spanning all course years and five course areas (Figure 2 *).* Students were aged between 18 and 32 (median 21), with 87% identifying as female, 10% as male, and 3% as non-binary or preferring not to report gender. These percentages are representative of gender distributions within participating HAU modules in the 2022–2023 academic year (e.g. 85% of 129 students in Principles of Animal Behaviour and Welfare and 85% of 54 students in Advances in Farm Animal Health, Welfare and Behaviour identified as female). Students from rural areas represented 55% of the sample, with 75% of these coming from non-farming backgrounds.

**Figure 3.**
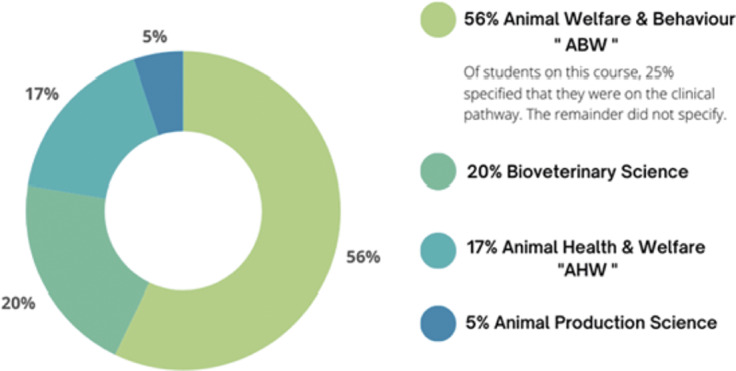
Distribution of students in Animal Science courses surveyed to assess UK undergraduate conceptions of animal welfare science

The Animal Science Course Area refers collectively to several course subtypes and the distribution of participating students within these groups is shown in Figure 3.

### Student knowledge of animal welfare

Students in different course groups differed in their level of agreement with the assertion “I already know everything there is to know about animal welfare” (Kruskal Wallis; χ^2^_(5)_ = 12.31; *P* = 0.031). ABW students were most likely to strongly disagree with this statement, significantly more (via Bonferroni-corrected Mann-Whitney *U* tests) than Vet Physio (*P* = 0.021) and Vet Nursing students (*P* = 0.015). The number of correctly identified freedoms varied by course (ANOVA; F_(5,116)_ = 3.9; *P* = 0.003), with ABW students able to identify more of the Five Freedoms than peers in Zoology (mean difference [MD] = 1.7; *P* < 0.05) and Wildlife Conservation (MD = 1.9; *P* < 0.05).

### Exploratory Factor Analysis

**Figure 4.**
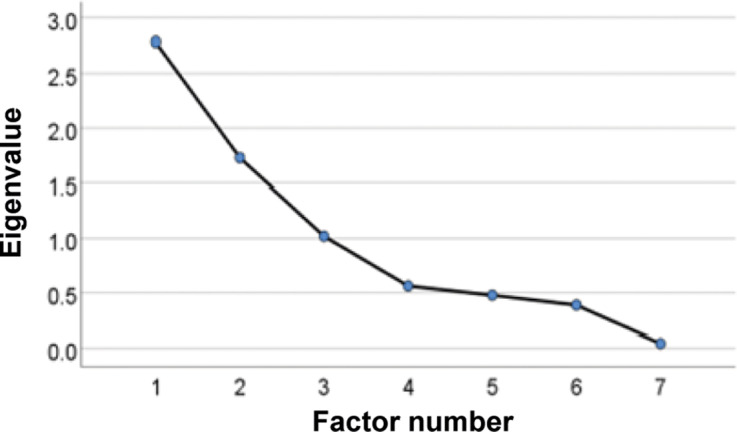
Scree Plot showing the eigenvalues (y-axis) for the first seven extracted factors (x-axis) in the Teaching Exploratory Factor Analysis, conducted to evaluate UK undergraduate student attitudes toward the teaching of animal welfare

Three factors were retained in the Teaching EFA, accounting for 79% of the total variance. (Figure 4, Table 2).

**Table 2.** Table listing the retained factors for the Teaching Exploratory Factor Analysis, their eigenvalues, the percentage of variance accounted for by each factor, and the cumulative variance after the addition of each factor

Factor	Eigenvalue	%Variance	Cumulative %
1	2.79	39.80	39.80
2	1.73	24.68	64.48
3	1.02	14.56	79.04

The final Varimax-rotated factor solution for the Teaching EFA is shown in Table 3.

**Table 3.** Final Varimax-rotated factor solution for the Teaching Exploratory Factor Analysis. Only items with a loading of > |.4| in at least 1 factor have been included in the final solution. The relevant loadings are represented in bold. The results from a goodness-of-fit test are also reported

Items	Factors		
	1	2	3
I am satisfied with how I have been taught animal welfare at my University	**0.97**	0.16	0.11
I am confident in my lecturers’ knowledge of animal welfare	**0.94**	0.13	0.19
Different animal welfare modules repeat the same information	**0.64**	–0.13	0.20
I have learned new information as a result of my animal welfare module(s)	**0.41**	**0.41**	–0.28
Some of my opinions have changed as a result of my animal welfare module(s)	0.19	**0.99**	0.05
Animal welfare is not as relevant to my course as other more specialised modules	0.08	**–0.40**	**0.59**
I have found the content in my animal welfare module(s) to be too difficult	0.16	0.11	**0.40**

Goodness-of-fit test satisfied: χ^2^_(3)_ = 1.66, *P* = 0.65.

In summary, Factor 1 (T_F1) represents satisfaction with the teaching of animal welfare, confidence in lecturers, acquisition of new information, and repetition of information. Factor 2 (T_F2) reflects the attitude that HAU animal welfare modules are relevant and have led to changes in opinion and acquisition of new information. Factor 3 (T_F3) represents the attitude that animal welfare content is both too difficult and not as relevant.

**Figure 5.**
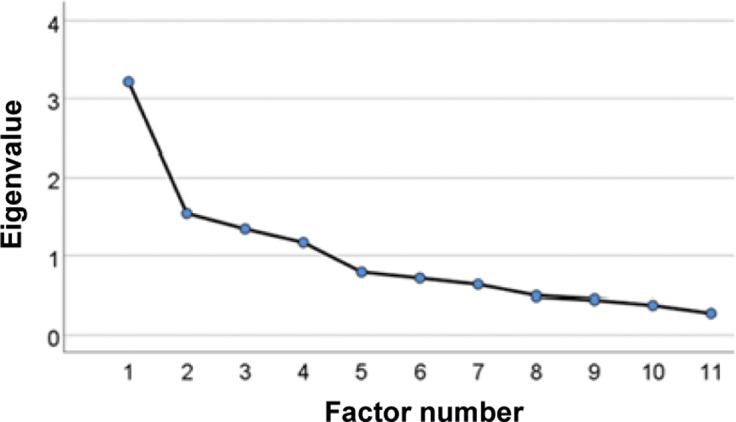
Scree Plot showing the eigenvalues (y-axis) for the first seven extracted factors (x-axis) in the Welfare Science Exploratory Factor Analysis, conducted to evaluate UK undergraduate student attitudes toward animal welfare and scientific fields in general.

Four factors were retained in the Welfare Science EFA; these accounted for 66% of the total variance (Figure 5, Table 4). The final Varimax-rotated factor solution for the Teaching EFA is shown in Table 5

**Table 4.** Table listing the retained factors for the Welfare Science Exploratory Factor Analysis, their eigenvalues, the percentage of variance accounted for by each factor, and the cumulative variance after the addition of each factor

Factor	Eigenvalue	% Variance	Cumulative %
1	3.22	29.23	29.23
2	1.54	14.03	43.26
3	1.34	12.22	55.48
4	1.17	10.63	66.11

**Table 5.** Final Varimax-rotated factor solution for the Welfare Science Exploratory Factor Analysis. Only items with a loading of > |.4| in at least 1 factor have been included in the final solution. The relevant loadings are represented in bold. The results from a goodness-of-fit test are also reported

Items	Factors			
	1	2	3	4
You shouldn’t bother trying to measure animal welfare because it is too subjective	**0.66**	0.19	*0.39*	0.03
Animal welfare is not a real science because everyone views it differently	**0.61**	0.17	0.06	–0.21
Animal welfare is a scientific discipline	**–0.59**	–0.13	–0.06	0.13
Animal welfare and animal rights are pretty much the same	**0.47**	0.28	0.05	–0.11
I already know all there is to know about animal welfare	**0.44**	–0.03	–0.08	0.13
The study of animal welfare is too straightforward to be interesting	0.13	**0.99**	0.04	–0.03
Animal welfare is an intellectually stimulating area of study	*–0.31*	**–0.63**	–0.09	0.19
I find science too difficult	–0.04	0.04	**0.75**	0.01
I find scientific modules intellectually stimulating	–0.17	–0.05	**–0.67**	0.21
Science does not need to be completely free of personal values	–0.03	–0.05	0.00	**0.63**
Good scientists acknowledge how personal values influence their work	–0.06	–0.09	–0.15	**0.60**

Goodness-of-fit test satisfied: χ^2^_(17)_ = 19.08, *P* = 0.32.

Factor 1 (F1) encompasses dismissive attitudes towards animal welfare, including the opinions that one should not bother measuring animal welfare, that there is nothing more to learn about it, that animal welfare is not a real science, and that it is the same as animal rights. Factor 2 (F2) reflects the views that animal welfare is too straightforward to be interesting and is not a stimulating area. Factor 3 (F3) reflects similar views as F2, but to science in general. The fourth and final factor (F4) pertains to student attitudes toward the influence of personal values in science.

### Linear models

Full linear model results are available in Tables S2–S6 in Supplementary material_1. In summary, students who found the study of animal welfare to be both too difficult and less relevant (T_F3) were more likely to be enrolled in Zoology (*P* = 0.009), Wildlife Conservation (*P* < 0.001), Vet Physio (*P* < 0.001), or other Animals courses (*P* = 0.002) compared to ABW. There was a negative relationship between Factor T_F2 (students learning new information and opinions changing because of animal welfare modules) and Factor T_F3 (students finding the content difficult; *P* < 0.001). Moreover, an interaction with T_F3 and gender suggested that this negative relationship was more pronounced in male students (*P* < 0.001).

ABW students found more relevance and opinion change from their animal welfare modules (T_F2) compared to Zoology (*P* = 0.005), Wildlife Conservation (*P* = 0.002), Vet Physio (*P* < 0.001), and Vet Nursing (*P* = 0.001). The Vet Nursing cohort expressed the most dismissive attitudes to the study of animal welfare (F1), significantly more than Wildlife Conservation (*P* = 0.002), ABW (*P* = 0.023), and Vet Physio (*P* = 0.016).

Student prioritisation of welfare spheres was associated with attitudinal factors: students who ranked natural living (*P* = 0.001) or affective state (*P* = 0.001) as the least important welfare sphere were more likely to find animal welfare modules difficult and less relevant (T_F3). Students prioritising natural living were also the least satisfied with the teaching of animal welfare at HAU (T_F1; *P* < 0.001). Despite these results, 84% of all participants expressed at least some satisfaction (Likert scores of ≥ 5) with the teaching of animal welfare science at HAU.

### Thematic analysis

Two distinct theme sets are described below, the first relating to the definitions of animal welfare provided by students, and the second relating to student perceptions of animal welfare as a science. A few quantitative results also directly support these results; for instance, select themes identified in the thematic analyses were followed up using inferential statistical tests. Thus, these results have also been included in this section to complement the findings of the qualitative synthesis.

#### Students’ animal welfare definitions

Students’ responses to the question “How would you define animal welfare?” were summarised under six main themes (see Table 6), as follows:

**Table 6.** Themes and corresponding subthemes (where applicable) identified in the thematic analysis of students’ animal welfare definitions. Themes are ordered from most to least frequent. Exemplar quotations are also presented

Themes	Subthemes	Exemplar quotations
Physical Health		*Health and physical well-being of an animal*
Emotional Health		*The emotional stability of the animal*
Resource-based criteria	Duty of care Human provision of resources or environment Unspecified “Needs” Unspecified resources	*Safeguarding animals from harm or suffering* *Ways in which humans can help to keep animals in the most realistic environment* *Ensuring all needs of animals are met* *The resources available to them*
Established conceptualisations	Coping Five Domains Quality of Life Three spheres	*How well the animal is coping with their current situation* *Using the 5 Domains* *The quality of life experienced by the animal* *I would use Frasers 3 spheres of welfare to define animal welfare. Where natural living, affective state and health and functioning of an animal are all just as important*
Tautology	Wellbeing Welfare Ethics	*Study of an animals* [sic] *well-being* *[animal welfare is] the pursuit of information to better assist the welfare of animals* *[animal welfare is] the ethics in animal welfare*
Behaviour	Behaviour in general Natural or normal behaviours	*Looking at what the animals require for good welfare and how this should be efficiently measured using a variety of behaviours.* *Whether an animal is given the opportunity to express its natural behaviours*

Since the entire sample of students responded to this question, we have also recorded the number of students whose definitions were coded into these themes and subthemes and note that the majority of definitions were coded into multiple themes. Of 123 responses, ‘physical health’ (45%) was the most commonly referenced theme. This finding was consistent with students’ ranking of ‘health and biological functioning’ as the most important welfare sphere overall (74%), compared to ‘affective [i.e. emotional] state’ (15%) and ‘natural living’ (11%). However, there did appear to be a difference in prioritisation according to student background: urban students typically ranked ‘affective state’ higher than ‘natural living’; the inverse was true for rural students (Fisher’s Exact Test; *P* = 0.036).

Students also mentioned animals’ ‘psychological’, ‘social’, or ‘emotional health’ (32%). ‘Behaviour’ was also frequently mentioned (19%), most often in relation to whether animals could express their ‘normal’ or ‘natural’ behaviour. The frequent reference to ‘physical health’ in students’ own welfare definitions is consistent with their prioritisation of this sphere.

Many students (22%) equated ‘welfare’ with ‘well-being’, e.g. “*Animal welfare can be essentially defined as the well-being of non-human animals”* (third-year Zoology). Others equated welfare with ethics (5%), and/or provided tautologous definitions of animal welfare that referenced animal welfare itself (3%), such as “[Animal welfare is] *a combination of animal health and welfare of animals”* (fourth-year Bioveterinary Science).

Some students provided established conceptualisations of welfare, referencing: the Five Freedoms and/or Five Needs (16%; Farm Animal Welfare Council [1979]; Animal Welfare Act [2006]); Quality of Life (13%; Green & Mellor [2011]); the three spheres (7%; Fraser *et al.* 1997); ‘Coping’ (5%; Broom [1996]); or the Five Domains (2%; Mellor & Reid [1994]). However, many students also defined animal welfare solely using resource-based criteria (which were often unspecified), framed in terms of human provision of resources to an animal, e.g. *“The conditions and state animals are kept in and how they are treated by humans”* (first-year Vet Physio) or “*The care and environment that animals need”* (third-year ABW). Such definitions often referenced animal needs (20%), the conditions or environment in which animals are kept (22%), and the duty to protect animals or treat them in a certain way (22%). Vet Nursing and Wildlife Conservation students were more likely than other cohorts to provide the definitions relating solely to resource-based criteria, rather than to the internal state of the animal (i.e. Theme 3 shown in Table 3; Chi-squared; χ^2^_(5)_ = 21.29; *P* = 0.001).

#### Animal welfare as a science

Forty-six students responded to the question “Feel free to tell us more about why you do or do not think animal welfare is a legitimate science”. We identified three overarching themes: animal welfare as a ‘hard science’; animal welfare as partly scientific; and animal welfare as a non-science (Table 7). Underlying these themes was a generalised assumption amongst the student body that whether animal welfare was seen as a legitimate scientific field was contingent on the extent to which it was perceived to rely upon methods drawn from the natural sciences.

**Table 7.** Themes and subthemes identified in response to the question “Feel free to tell us more about why you do or do not think animal welfare is a legitimate science.” An exemplar quotation is also included to illustrate each subtheme

Overarching theme	Subthemes	Short exemplar quotations
Animal welfare as a ‘hard science’	Because of biological and chemical processes Because of data and ‘facts’	*Requires an understanding of brain chemistry* *It is based on scientific facts*
Animal welfare as partly scientific	Because it involves science Because of ‘hard science’ elements Because it is new	*There is some science base in it* *It’s partly scientific as of the genetics* *More research is required to make it more scientific*
Animal welfare as a non-science	Because of subjectivity Because of ethics Because animal welfare is something else	*It takes a degree of subjectivity* *It’s more of an ethical discipline* *Some people believe it’s a general discipline.*

##### Animal welfare as a ‘hard’ science (37% of the 46 students supplying free-text comments)

Proponents of animal welfare as a science (i.e. students who either agreed or strongly agreed that animal welfare was a science) invoked features of the discipline that aligned with the physical and biological sciences. Such views were evident amongst students on welfare-focused courses (e.g. “*To study welfare thoroughly you need to have a basis in animal biology which no one would argue* [isn’t] *scientific”*[third-year AHW] and “*Science is used to determine animal welfare biologically and chemically within them”* [second-year ABW]) as well as those in veterinary disciplines (e.g. *“Areas like the endocrine or neurological systems work alongside animal welfare”* [second-year Vet Nursing] and “*…welfare is related to many physiological components such as cortisol levels being indicative of stress”* [fourth-year Bioveterinary Science]).

Consistent with Shapin’s (2022) assertions regarding perceptions of ‘science’, students who viewed animal welfare as a science typically referenced The Scientific Method, measurements, and facts: *“Research is carried out and scientific methods are used to analyse data determining the facts”* [fourth-year ABW] or “*It is something that can be observed and measured. These observations can be recorded and analysed thus making it possible for credible scientific studies to be produced”* [third-year Zoology].

##### Animal welfare as partly scientific (33% of the 46 students supplying free-text comments)

Other students considered animal welfare to partly, but not fully, meet the threshold of a ‘true’ scientific discipline. Such students tended to make conditional statements that suggested that animal welfare was promising but not ‘fully there’ as a science. This view was particularly evident amongst students on veterinary-focused courses; for example, students shared that: “*More research is required to make it more scientific, however it is heading in the right direction*” [second-year Vet Physio], *“I think the science behind the anatomy and physiology… is somewhat scientific”* [fourth-year Vet Nursing], and *“Yes it has some science, but a lot of it is also opinion, theories etc”* [second-year Vet Nursing]. Thus, these students seemed to recognise, quite rightly, that animal welfare extended beyond the biological sciences; however, this deviation was seen to weaken the scientific promise of the discipline.

##### Animal welfare as a non-science (28% of the 46 students supplying free-text comments)

Finally, a portion of students held that they did not consider animal welfare to be a science at all. Here, the element of subjectivity and the influence of individual opinions represented the most common subtheme among dissenters of animal welfare as a science (i.e. those who disagreed or strongly disagreed with the statement “animal welfare is a scientific discipline”). Specifically, the words ‘subjectivity’ or variants thereof (e.g. ‘subjective’, ‘not objective’) appeared in 62% of the definitions within this theme. Students shared, for example, that animal welfare was “*Very subjective in areas… A lot of holistic and opinion approaches”* [second-year Vet Nursing] or that *“The value we put on animals’ worth is subjective … making the study of animal welfare less sciencey”* [fourth-year Vet Physio].

Such statements seem to reflect a positivist epistemology of value-free science (Slevitch 2011). Indeed, several students provided viewpoints on the relationship between science and the discipline of animal ethics, casting doubt on animal welfare as a scientific discipline due to its relationship with ethical thinking (e.g. “*In my opinion animal welfare is probably more of an ethical discipline”* [third-year AHW]; *“…it cannot exist without emotion attached which means welfare issues are often approached in a less objective way”*[fourth-year Vet Nursing]; and “*The discipline seems more buried in morality and ethics”* [fourth-year Vet Nursing]). That the concern for animal welfare is guided by ethical reflections or social demand, does not make animal welfare science any less scientific. However, some of these statements also suggest that students view the field of animal welfare as more susceptible to poor scientific practice due to its more obvious connections to value systems.

Others, even several in ABW courses, were more direct in asserting that animal welfare was entirely distinct from their conception of ‘science’. Here, comments included, “*It isn’t really a science I think it is more of a pseudoscience if anything, since it is about laws and behaviour”* [first-year ABW] and *“I feel more strongly inclined to suggest animal welfare…is more so a service industry*” [third-year ABW].

## Discussion

This mixed-methods study sought to identify conceptions and attitudes related to animal welfare as a science amongst undergraduate students pursuing animal-focused courses at a UK university.

### How students define and understand animal welfare

To our knowledge, this study is one of the first to evaluate which sphere of welfare is most important to undergraduates in animal-centric pathways of study. Most students (74%) indicated they prioritised the ‘health and biological functioning’ of Fraser *et al.* (1997)’s three spheres and by extension, ‘physical health’ was the most common theme referenced when articulating their own welfare definitions. This welfare sphere also receives disproportionate attention from the scientific research community (Beaver *et al.* 2021 and is prioritised among stakeholders with farming backgrounds (Kauppinen *et al.* 2010), animal scientists, and veterinarians (Sumner & von Keyserlingk 2018). Many of these stakeholder groups are engaged in teaching at HAU, which may provide a partial explanation for students’ focus on and prioritisation of this sphere. Of the three spheres, health and biological functioning arguably lends itself most easily to scientific methods aligning with the ‘hard sciences’; thus, students’ broader views toward the meaning of science may also contribute to this focus.

Only 15% of students ranked ‘affective state’ as most important for animals. In contrast, a relatively recent study amongst veterinary students in the United States (Proudfoot & Ventura 2021) observed that the highest percentage of students prioritised affective state over the other spheres. Whether these contrasting findings are better explained by individual variation, timing and degree of exposure to differing welfare curricula and messaging, or to geographic and/or cultural differences is unclear and is an area meriting further study. It is worth noting that in the present study nearly one-third of students raised issues related to affective state (e.g. psychological, social, and emotional health of animals) in their qualitative definitions of animal welfare, suggesting that considerations related to the mental state of animals did actually feature in a proportion of students’ conceptions of welfare. Natural living was the least frequently prioritised, with just 11% of students ranking this sphere as most important to animal welfare. However, rural students had a greater appreciation for ‘natural living’ compared to urban students, a finding that complements prior research into rural dwellers’ strong affective connections to the natural environment (Kals *et al.* 1999; Hinds & Sparks 2008).

Adequate resources play a critical role in animals’ ability to cope, but resources alone cannot ensure positive welfare (Rushen *et al.* 2011). Here, Wildlife Conservation students were more likely to define animal welfare on the basis of resources provided by humans. This external perspective may relate to the conservationist focus on human impacts on wildlife populations (Crowley *et al.* 2022): the “*suffering wildlife endures because of humans*” is seen as a shared responsibility (Paquet & Darimont 2010; p 177). Vet Nursing students were similarly more likely to define animal welfare based upon resources and appropriate treatment of animals by humans. This perspective may be influenced by an ethos of a strong duty of care (RCVS; Orpet & Welsh 2010) and their significant role in educating clients on upholding positive animal welfare (Ackerman 2012).

### The perceived difficulty of animal welfare science

We report a strong link between low student interest in animal welfare as a topic of study and high perceived difficulty. More research is needed to determine the extent to which this relationship is causal, and in which direction. A lack of interest may lead to low engagement, making the content seem more difficult. Conversely, students finding the topic difficult may be less likely to engage. Bowman (2017) clarifies how *both* inherent interest and perceived ability are key components of motivation and self-regulated learning. Due to the entwined nature of ability, interest, and motivation, there is likely to be a bi-directional relationship between perceiving a topic to be difficult and perceiving it to be unstimulating, creating a cycle of reinforcement (MacLellan 2008).

Not surprisingly, students who found their animal welfare content difficult were also less likely to learn new information or experience changing opinions over the course of their modules. However, this pattern was unexpectedly stronger in male students. This finding is perhaps out of alignment with the observations of Kardash and Wallace (2001), who found that male (compared to female) university students felt their science instructors took time to explain difficult material so that students could acquire new information and understanding. Although the gender distribution in our study was representative of the spread within the selected modules, 87% of our students identified as female, so we contend that the comparatively small percentage of students of other genders may not allow for a sophisticated exploration into gender differences. As elucidated by Taasoobshirazi and Carr (2008), gender differences in science must be examined using a multifactorial lens, taking into consideration the interaction between motivation, cognition, and social factors.

In their seminal work, Kruger and Dunning (1999) reported that participants scoring in the lowest quartile in various tests were most likely to overestimate ability. This phenomenon has achieved high replicability in social psychology (Mazor & Fleming 2021), although multiple theories may explain its origin (Jansen *et al.* 2021). In the present study, students who reported that they already knew everything about animal welfare were able to identify fewer of the Five Freedoms and were more likely to misidentify animal welfare as ‘the same’ as animal rights. In contrast, ABW students scored the lowest on the dimension “I already know everything there is to know about animal welfare” despite being able to identify the most freedoms. However, the extent to which the Dunning-Kruger effect may account for these findings is unclear, as other recent research has called into question the legitimacy of the phenomenon (Gignac & Zajenkowski 2020, 2023; Lebuda *et al.* 2024). Other possible explanations include that inherent interest in the topic may correlate with openness to learn (Ziegler *et al.* 2018); indeed, ABW students also reported the highest opinion change following their animal welfare modules.

### Student satisfaction with the teaching of animal welfare

Interestingly, students who prioritised natural living expressed the most dissatisfaction with how animal welfare was taught at HAU. This may be due, in part, to the welfarist focus on intensive animal systems (Paquet & Darimont 2010). Indeed, the origins of animal welfare science stem from concerns regarding the intensification of farming systems, with the behaviour of wild counterparts provided as a juxtaposition (Harrison 1964; Brambell 1965). These topics feature in many introductory animal welfare modules, and it is possible that students find welfare concerns over animals in more naturalistic settings to be discounted. In future, interviews with students from rural backgrounds may allow us to understand the origin of this impasse and to ensure that the values of this group are not overlooked.

The Vet Nursing cohort expressed the most dismissive attitudes to animal welfare science, as operationalised through factor analysis. Indeed, Johnstone *et al.* (2019) found that students entering the veterinary profession rationalised that they had already received welfare education throughout their training, so could “*figure this out on my own*” (p 7). These negative perspectives on animal welfare science curricula may arise when students consider animal welfare to already be integrated into modules they are taking as part of their veterinary education (Mijares *et al.* 2021).

Despite these findings, most students (84%) expressed at least some satisfaction with the teaching of animal welfare science at HAU. A number of students also highlighted the repetitiveness of animal welfare material across different modules. Encouragingly, repetition (specifically the statement “Different animal welfare modules repeat the same information”) was not associated with dissatisfaction. In fact, it was weighted in the same direction as ‘confidence in lecturers’ and ‘acquisition of new information’ (see Table 3). Studies across several research disciplines have also outlined the benefit of spaced repetition on long-term conceptual memory (Voice & Stirton 2020) and on self-assessments of learning (Logan *et al.* 2012).

### Student views on animal welfare as a science

All three main themes within the thematic analysis suggest that students tended to equate ‘science’ with the natural sciences and dismiss the social sciences as subjective and hence ‘unscientific’. Student dissenters of animal welfare as a science seemed to recognise the discipline as spanning beyond the natural sciences, which demonstrates nuanced understanding; however, rather than viewing the integration of ethics and the social sciences as a strength, this was seen to diminish the scientific power of inquiry. Thus, these views are consistent with Shapin’s (2022) perspective on the differential apportioning of value between the so-called ‘hard’ and ‘soft’ sciences.

In contrast to these viewpoints, Fraser *et al.* (1997) cautions against attempts to distance welfare science from ethics. Rather, the authors highlight that as a societally mandated science, animal welfare science does, and should (like many other fields), reflect ethical concerns to orient research towards priority areas and appropriately interpret findings. Animal welfare science spans a multidisciplinary integration of both natural and social sciences (Marchant-Forde 2015). While animal welfare science and ethics are distinct disciplines, they also intersect, and animal welfare questions can often be best pursued by integration of methods from both the natural and social sciences. Fundamentally, social science methods can be harnessed towards understanding ethical viewpoints of stakeholders. As Hoffding (1905) writes, “*Ethical ideals and ethical endeavors… are objects of sociological research*” (p 177). Thus, social science is likely to be foundational in helping the findings generated from the more ‘natural’ side of animal welfare science achieve implementation in the real world, by leveraging understanding of human and societal systems to realise change (Ventura & Fjæran 2021).

### Future directions and recommendations for action

Here, we enumerate three directions for future action research with the goal of improving the provision of, and appreciation for, animal welfare curricula at the undergraduate level:

#### Increased engagement with dissatisfied student groups

The current study revealed that students prioritising natural living were the most dissatisfied with the provision of animal welfare science curricula in this UK university environment. Importantly, in-depth qualitative work is needed to engage with such student subgroups, even if they do not represent majority opinion. Nielsen and Lyhne (2016) describe a methodological approach to action-oriented interviews as an “*area for generating action through mutual reflection*” (p 54). A goal of such interviews is to create collective learning experiences for participants and researchers, and to empower participants to contribute to action.

As the veterinary nursing cohort expressed more dismissive attitudes towards animal welfare science compared to other course groups, these students should also be invited to focus groups or interviews to share their lived experiences and openly discuss priorities (Lloyd-Evans *et al.* 2023). Yeates (2014) discusses the vital role of the veterinary nurse in advancing animal welfare, including, at times, acting as the consciences of both owners and veterinarians. Within veterinary nursing courses in the UK, it is possible there may be a disconnect between concepts of animal welfare taught within veterinary nursing modules, and those that are put forward in welfare-specific modules open to a range of course groups. Communication between lecturers in both types of modules is essential to understand the broader picture of the students’ course.

#### Elevation of the social sciences in animal welfare curricula

Students in our study equated ‘science’ with ‘natural sciences’ and many implied that animal welfare investigations driven by the social sciences were less scientific, rigorous, or valuable. This viewpoint tends to be mirrored by the wider academic community (Chompalov & Popov 2021). Appreciation for, and understanding of, the social sciences must be embedded into the broader curriculum as well as within specific animal welfare modules. Lund *et al.*(2006) articulated the challenges of integrating multiple disciplines by acknowledging that *“each disciplinary community has a different way of speaking about the topics and conduct of its research. The problems lie not only in the technical terminology, but also the manner in which information gains credibility*…” (p 43). However, despite these challenges, Lund *et al.*(2006) recommend that interdisciplinary ways of working, including integration of the social sciences, should begin in undergraduate education. The positivist view of a value-free science appears at odds with interdisciplinary thinking, since positivism tends to downplay the merits of social science (Chompalov & Popov 2021). Techniques such as frame reflection may be beneficial to help students learn “…*to parse how and why someone’s stance may by influenced by values and by science*” (Proudfoot & Ventura 2021; p 368).

#### Improved understanding of lecturer attitudes

Tzioumis *et al.* (2018) uncovered a variety of educator attitudes towards the veterinary animal welfare curriculum. For instance, male educators (in Oceania) were more likely to emphasise the importance of ‘the development of animal welfare science,’ whereas female educators more often prioritised ‘applied animal ethics.’ The attitudes of animal welfare educators are likely to influence the learning and viewpoints of their students (Heleski *et al.* 2005; Izmirli & Phillips 2012; Tzioumis *et al.* 2018); however, at present, it is unknown exactly how this influence may be manifested. In the current study, students grappled with the relationship between animal welfare science and ethics, and the role of the social sciences in mediating this relationship. By administering a similar (appropriately modified) questionnaire to lecturers, we may be able to determine the extent to which lecturer attitudes and epistemologies exert influence on student perception. This diversity in educator perspectives can be valuable for providing students with a rich, multifaceted understanding of animal welfare, and more research is needed to understand how these perspectives shape student learning and the field as a whole.

Most students in our study prioritised the sphere of biological health and functioning, which historically has also been emphasised by veterinary and agricultural stakeholders (Kauppinen *et al.* 2010; Sumner & von Keyserlingk 2018). These stakeholder groups often play a key role in delivery of taught material to students in animal-centred courses, which may catalyse the construction of student views or reinforce their existing attitudes. Further research is needed to explore the attitudes of animal welfare educators beyond the veterinary context, both within and outside the university population studied here.

### Study limitations

Compared to urban students, rural students in our study placed more emphasis on the welfare sphere concerning ‘natural living’. This finding conflicts with a body of research suggesting that individuals from rural farming backgrounds place less emphasis on ‘natural living’ compared to other spheres (e.g. Vanhonacker *et al.* 2008; Benard and de Cock Buning 2013; Spooner *et al.* 2014). Our result may stem from a comparatively small proportion of rural students from farming backgrounds in our study (n = 17). Previous research also suggests that an organic vs conventional farming background may influence the perceived importance of naturalness to animal welfare (see Lund 2006), a nuance that we were not able to capture in the present study. Thus, future work should aim to understand the viewpoints of a larger group of students from a variety of farming backgrounds.

In addition, the next stages of research should aim to provide representative distributions across courses and years, as Vet Physio (37%) and second-year (39%) students were overrepresented in the current sample. Modest monetary incentivisation may provide the opportunity to bolster representation (Mercer *et al.* 2015), although this may present challenges within student populations. Alternatively, students could be given credit towards their modules for simple completion of the survey (with responses anonymised), which may allow for the questionnaire to be lengthened to further validate measures under study; the consideration of power-distance relationships in this case would be paramount (Leentjens & Levenson 2013). As supported in similar research (e.g. Kind *et al.* 2007), our questionnaire was kept brief to avoid burdening students with lengthy response times, but this decision came at the expense of evaluating concurrent validity. Further, some of the open response questions were marked as optional, again to encourage completion of the questionnaire. Questionnaires in general are susceptible to non-response bias (Sedgwick 2014), and this may be compounded by the provision of optional questions to which a certain subtype of student may be more inclined to respond.

Limitations of exploratory factor analysis as a method also warrant discussion. EFA has been described as simultaneously quirky, temperamental, valuable, and interesting (Osborne & Fitzpatrick 2012). It is a useful procedure for variable reduction, but the process hinges upon decisions regarding factor extraction and rotation methods, which can be subjective (Reio Jr & Shuck 2015). Bias may also arise in the researchers’ interpretation and designation of factor loadings. We aimed to reduce bias in our analysis by adhering to guidelines regarding item-to-subject ratios and minimum sample size (de Winter *et al.* 2009) and ensuring that assumptions were not violated (e.g. by transforming all variables and assessing goodness-of-fit). We also input items into our EFA dataset using question numbers rather than text descriptions of the items, referring back to the original Likert-scale questions only after a factor solution was determined. Although EFA has been reported to suffer from poor replicability (Costello & Osborne 2005), the aim of our work as action research centres around understanding of a specific context. Future work could ensure that the factors are indeed representative of their relevant constructs, perhaps through the use of an expert panel (as suggested in Burny & Leiva 2023).

Finally, this study focused on students at a single UK University. This small population of interest should not be considered a limitation, as generalisation to other populations is not an established aim of action research (Thompson & Perry 2004). However, conclusions can inform broader investigations into attitudes towards animal welfare science in UK higher education and elsewhere.

### Animal welfare implications

Undergraduate students in animal-centred courses will go on to assume a wide variety of roles with animals, including veterinary nurses, animal behaviourists, research scientists, and wildlife conservationists. An understanding of, and respect for, animal welfare science is paramount for the next generation of professionals and the future animals in their care. This work is one of the first to explore the attitudes of undergraduate students towards animal welfare *as a science* and paves the way for future research in this area. As educators in animal welfare, we hope this research may ultimately contribute to improvements in teaching provision of this valuable scientific discipline.

## Supporting information

Beaver and Ventura supplementary material 1 Beaver and Ventura supplementary material

Beaver and Ventura supplementary material 2 Beaver and Ventura supplementary material

## Supplementary material

The supplementary material for this article can be found at http://doi.org/10.1017/awf.2025.10032.
