# Supplementary material for: Undergraduate student attitudes towards animal welfare science: An investigation to inform teaching approaches
Source: Anim Welf. 2025 Aug 19;34:e58. doi: 10.1017/awf.2025.10032 (PMC12451403; doi:10.1017/awf.2025.10032)
Supplement: Beaver and Ventura supplementary material 1 — Beaver and Ventura supplementary material [file S0962728625100328sup001.pdf]

Undergraduate student attitudes towards animal welfare science: An investigation to inform teaching approaches

Annabelle Beaver[1] and Beth A Ventura[2, 3]

[1] Animal Behaviour and Welfare Research Group, Animal Science Research Centre, Harper Adams University, Newport, Shropshire TF10 8NB, UK

[2] Large Animal Clinical Sciences, College of Veterinary Medicine, Michigan State University, East Lansing, MI, USA

[3] Department of Life Sciences, University of Lincoln, Brayford Pool, Lincoln LN6 7TS, UK

* Author for correspondence: Annabelle Beaver, email: abeaver@harper-adams.ac.uk

Supplementary material_1

Table S1 *A priori* research questions for the inferential statistical analysis.

1. Do students in different course groups differ in their beliefs that animal welfare is a legitimate science?
2. Do students in different course groups differ in their knowledge of animal welfare (using Five Freedoms as a proxy) or in their *perceived* amount of knowledge of animal welfare?
3. Do students in different course groups differ in the sphere of welfare they prioritise (i.e., health and biological functioning, affective state, or natural living)
4. Is the sphere of welfare prioritised by students related to student background (i.e. rural or town/city)?
5. Are any latent factors influenced by student background (i.e. rural or town/city), welfare sphere prioritised, gender, or course group?

Tables showing the full results of linear models are presented in Supplementary Tables S2–S6. Due to the backwards stepwise process of predictor variable removal, models without any variables meeting the $P < 0.05$ threshold have not been included.

Table S2. Final model results for the general linear model with Factor T_F2 (students learning new information and opinions changing as a result of animal welfare modules) as the outcome. Slope estimates, standard errors of the estimates, *t*-values, and associated *P*-values are shown.

Parameter	Estimate	Standard Error	*t*-value	*P*r > |t|
Intercept	0.75	0.18	4.10	<0.001
Other Animal Science courses	-0.28	0.29	-0.97	0.335
Veterinary Nursing	-1.01	0.30	-3.35	0.001
Veterinary Physiotherapy	-1.13	0.23	-5.01	<0.001
Wildlife Conservation	-1.15	0.37	-3.14	0.002
Zoology	-0.87	0.31	-2.83	0.005
Animal Behaviour & Welfare	Reference	-	-	-

Table S3. Final model results for the general linear model with Factor F1 (dismissive attitudes towards animal welfare) as the outcome. Slope estimates, standard errors of the estimates, *t*-values, and associated *P*-values are shown.

Parameter	Estimate	Standard Error	*t*-value	*P*r > |t|
Intercept	0.56	0.22	2.50	0.014
Animal Behaviour & Welfare	-0.65	0.28	-2.31	0.023
Other Animal Science courses	-0.42	0.30	-1.39	0.167
Veterinary Physiotherapy	-0.63	0.25	-2.46	0.016
Wildlife Conservation	-1.14	0.37	-3.10	0.002
Zoology	-0.56	0.32	-1.74	0.085
Veterinary Nursing	Reference	-	-	-

Table S4. Final model results for the general linear model with Factor T_F3 (Animal welfare is difficult and not relevant) as the outcome. Slope estimates, standard errors of the estimates, *t*-values, and associated *P*-values are shown.

Parameter	Estimate	Standard Error	*t*-value	*P*r > |t|
Intercept	0.21	0.24	0.87	0.386
Factor T_F2	-1.25	0.27	-4.61	<0.001
Gender (Male vs Female)	-0.27	0.25	-1.07	0.29
Factor T_F2 x Gender (Male vs Female)	1.24	0.28	4.42	<0.001

Table S5. Final model results for the general linear model with Factor T_F1 (satisfaction with the teaching of animal welfare) as the outcome. Slope estimates, standard errors of the estimates, *t*-values, and associated *P*-values are shown.

Parameter	Estimate	Standard Error	*t*-value	*P*r > |t|
Intercept	-0.80	0.27	-2.96	0.004
Sphere of welfare prioritised:				
Affective state	0.94	0.35	2.70	0.008
Health & biological functioning	0.88	0.29	3.04	0.003
Natural living	Reference	-	-	

Table S6. Final model results for the general linear model with Factor T_F3 (animal welfare modules are difficult and not stimulating) as the outcome. Slope estimates, standard errors of the estimates, *t*-values, and associated *P* values are shown.

Parameter	Estimate	Standard Error	*t*-value	*P*r > |t|
Intercept	-0.39	0.16	-2.38	0.019
Sphere of welfare prioritised:				
Health & biological functioning	-1.30	0.38	-3.37	0.001
Natural living	-0.04	0.12	-0.36	0.723
Affective state	Reference	-	-	-
Course Group:				
Other Animal Science courses	0.69	0.21	3.19	0.002
Veterinary Nursing	0.15	0.22	0.71	0.480
Veterinary Physiotherapy	0.93	0.17	5.58	<0.001
Wildlife Conservation	0.97	0.26	3.70	<0.001
Zoology	0.60	0.22	2.67	0.009
Animal Behaviour & Welfare	Reference	-	-	-
Rural vs. Urban background	-0.26	0.13	-2.07	0.041
