# Supplementary material for: Undergraduate student attitudes towards animal welfare science: An investigation to inform teaching approaches
Source: Anim Welf. 2025 Aug 19;34:e58. doi: 10.1017/awf.2025.10032 (PMC12451403; doi:10.1017/awf.2025.10032)
Supplement: Beaver and Ventura supplementary material 2 — Beaver and Ventura supplementary material [file S0962728625100328sup002.pdf]

Student Attitudes towards Animal Welfare

Page 1: Informed Consent Statement

Title of Study: How student perception of animal welfare influences teaching approaches

Principal Investigator: Dr Annabelle Beaver abeaver@harper-adams.ac.uk

Purpose of Study: You are being asked if you would like to participate in a research study, which will involve completion of a short survey. Before you decide if you'd like to participate, it is important to understand why the research is being done.

This survey is being conducted to gain more information about your perspectives and awareness of animal welfare. Ultimately, we would like to consider the student voice on these topics to improve the teaching of animal welfare at Harper Adams and beyond.

Confidentiality and Voluntary Participation: Although we would be grateful if you'd consider completing this survey, participation is completely voluntary. Participation or non-participation will not affect your mark in any module or your relationship with the researchers.

Your responses will be anonymous- there will be no way of checking whether you completed the survey. Because responses will be anonymous, we won't be able to identify individual surveys after submission. This means you won't be able to ask for your responses to be withdrawn after you've submitted them. However, you are welcome to exit the survey at any time before submission and your responses will not be counted.

Please be aware that there is no direct benefit to you for your participation in this study. However, by participating, you will be contributing to scholarly activity at the University. It is hoped the information obtained from this study may improve teaching of animal welfare at Harper Adams and other institutions in the future. Student participation is key to achieving this aim.

Consent: To participate in the survey, click "Next". By clicking this button you are confirming that

- I voluntarily agree to take part in this study
- I have read and understand the provided information
- I understand that the survey is anonymous
- I understand my participation is voluntary and that I am free to withdraw at any time before

submitting the survey without giving a reason and without consequence

Page 2

1. Please select the option that best corresponds to your level of agreement with this statement:

	Strongly disagree	Disagree	Somewhat disagree	Neutral	Somewhat agree	Agree	Strongly agree
Animal welfare is a scientific discipline	☐	☐	☐	☐	☐	☐	☐

2. Feel free to tell us more about why you do or do not think animal welfare is a legitimate scientific discipline *Optional*

Page 3

3. Please select the option that best corresponds to your level of agreement with each of the following statements:

	Strongly disagree	Disagree	Somewhat disagree	Neutral	Somewhat agree	Agree	Strongly agree
I find science too difficult	☐	☐	☐	☐	☐	☐	☐
I find scientific modules intellectually stimulating	☐	☐	☐	☐	☐	☐	☐
Science should be completely objective	☐	☐	☐	☐	☐	☐	☐
Science does not need to be completely free of personal values	☐	☐	☐	☐	☐	☐	☐
Good scientists acknowledge how personal values influence their work	☐	☐	☐	☐	☐	☐	☐

Animal welfare is not a real science because everyone views it differently	☐	☐	☐	☐	☐	☐	☐
Animal welfare and animal rights are pretty much the same	☐	☐	☐	☐	☐	☐	☐
You shouldn’t bother trying to measure animal welfare because it is too subjective	☐	☐	☐	☐	☐	☐	☐

Page 4

4. Please select the option that best corresponds to your level of agreement with each of the following statements:

	Strongly disagree	Disagree	Somewhat disagree	Neutral	Somewhat agree	Agree	Strongly agree
Animal welfare is not as relevant to my course as other more specialised modules	☐	☐	☐	☐	☐	☐	☐
The study of animal welfare is too straightforward to be interesting	☐	☐	☐	☐	☐	☐	☐
Animal welfare is an intellectually stimulating area of study	☐	☐	☐	☐	☐	☐	☐
I already know all there is to know about animal welfare	☐	☐	☐	☐	☐	☐	☐
I have found the content in my animal welfare module(s) to be too difficult	☐	☐	☐	☐	☐	☐	☐

I have learned new information as a result of my animal welfare module(s)	☐	☐	☐	☐	☐	☐	☐
Some of my opinions have changed as a result of my animal welfare module(s)	☐	☐	☐	☐	☐	☐	☐
I am confident in my lecturers' knowledge of animal welfare	☐	☐	☐	☐	☐	☐	☐
Different lecturers define animal welfare differently	☐	☐	☐	☐	☐	☐	☐
Different animal welfare modules mostly just repeat the same information	☐	☐	☐	☐	☐	☐	☐
I am satisfied with how I have been taught animal welfare at my University	☐	☐	☐	☐	☐	☐	☐

Page 5: Qualitative questions

5. How would you define animal welfare?

6. Animal Welfare is sometimes conceptualised in terms of 3 overlapping circles: Basic health and biological functioning (is the animal in good physical health?); Natural living (can the animal express highly motivated behaviours and lead a resonably natural life?); and Affective state (how does the animal feel?). Please rank the 3 spheres from most to least important for good animal welfare:

	Least important	Intermediate	Most important
Basic health and biological functioning	☐	☐	☐
Natural living	☐	☐	☐
Affective state	☐	☐	☐

7. Can you remember any of the 5 freedoms off the top of your head?

- ○ Yes
- ○ I cannot remember any right now

7.a. If you selected "Yes", please list the ones you can remember here:

8. In your experience, what, if anything, should be improved about how animal welfare is taught at Harper Adams? *Optional*

9. In your experience, what, if anything, has been effective about how animal welfare is taught at Harper Adams? *Optional*

10. What else would you like us to know about your experiences with learning about animal welfare at Harper Adams, or your thoughts on animal welfare in general? *Optional*

Page 6: Demographics

Almost finished! Please let us know a bit about you:

11. What course are you in?

12. What year of your course are you currently in?

- First year
- Second year
- Placement year
- Final year
- Other

12.a. If you selected Other, please specify:

13. What is your age? *Optional*

Please enter a whole number (integer).

14. How do you identify?

- a) Female

- b) Male
- c) Non-binary
- d) Prefer to self-identify
- e) Prefer not to say

14.a. If you selected "prefer to self-identify", please specify:

15. Including the module(s) you are currently enrolled in, how many welfare modules have you taken? (Include modules that mention animal welfare, even if they also address e.g., health or behaviour)

Please enter a whole number (integer).

16. Which best describes the type of environment in which you grew up?

- Rural, but not on a farm
- Rural, on a farm
- Town or city

16.a. What type of farm did you grow up on?

17. What types of animal(s) are you most hoping to work with after graduation?

Page 7: Final page

Thank you for taking the time to complete this survey! We are very grateful for your participation. If you have any questions, please do not hesitate to contact Dr Annabelle Beaver at abeaver@harper-adams.ac.uk

---
